# Predictors of hypoxemia in type-B acute aortic syndrome: a retrospective study

**DOI:** 10.1038/s41598-021-02886-9

**Published:** 2021-12-03

**Authors:** Yudai Tamura, Yuichi Tamura, Motoko Kametani, Yoshiaki Minami, Tomoko Nakayama, Daisuke Takagi, Takashi Unoki, Megumi Yamamuro, Akio Kawamura, Tomohiro Sakamoto, Tadashi Sawamura

**Affiliations:** 1 Division of Cardiology, Saiseikai Kumamoto Hospital Cardiovascular Center, 5-3-1 Chikami Minami-ku, Kumamoto, 861-4193 Japan; 2 Intensive Care Unit, Saiseikai Kumamoto Hospital Cardiovascular Center, 5-3-1 Chikami Minami-ku, Kumamoto, 861-4193 Japan; 3 Department of Cardiology, International University of Health and Welfare School of Medicine, 4-3 Kodunomori, Narita, Chiba, 286-8686 Japan

**Keywords:** Cardiology, Risk factors

## Abstract

Acute aortic syndrome (AAS) can be life-threatening owing to a variety of complications, and it is managed in the intensive care unit (ICU). Although Stanford type-B AAS may involve hypoxemia, its predictors are not yet clearly understood. We studied clinical factors and imaging parameters for predicting hypoxemia after the onset of type-B AAS. We retrospectively analyzed patients diagnosed with type-B AAS in our hospital between January 2012 and April 2020. We defined hypoxemia as PaO_2_/FiO_2_ ≤ 200 within 7 days after AAS onset and used logistic regression analysis to evaluate prognostic factors for hypoxemia. We analyzed 224 consecutive patients (140 males, mean age 70 ± 14 years) from a total cohort of 267 patients. Among these, 53 (23.7%) had hypoxemia. The hypoxemia group had longer ICU and hospital stays compared with the non-hypoxemia group (median 20 vs. 16 days, respectively; *p* = 0.039 and median 7 vs. 5 days, respectively; *p* < 0.001). Male sex (odds ratio [OR] 2.87; 95% confidence interval [CI] 1.24–6.63; *p* = 0.014), obesity (OR 2.36; 95% CI 1.13–4.97; *p* = 0.023), patent false lumen (OR 2.33; 95% CI 1.09–4.99; *p* = 0.029), and high D-dimer level (OR 1.01; 95% CI 1.00–1.02; *p* = 0.047) were independently associated with hypoxemia by multivariate logistic analysis. This study showed a significant difference in duration of ICU and hospital stays between patients with and without hypoxemia. Furthermore, male sex, obesity, patent false lumen, and high D-dimer level may be significantly associated with hypoxemia in patients with type-B AAS.

## Introduction

Acute aortic syndrome (AAS) is a serious disease that can be fatal and requires strict management in the intensive care unit (ICU). Although the prognosis of Stanford type-B AAS is better than that of Stanford type-A^1,2^, complications such as rupture or re-dissection of the aortic dissection and malperfusion of the renal, visceral, spinal, or low extremity vasculature could occur^1,3^. In addition, hypoxemia is known to occur after the onset of AAS, and this phenomenon is observed in both Stanford type-A and type-B AAS^4–8^. Although the mechanism underlying hypoxemia that occurs after AAS has not yet been elucidated, it has been reported that the durations of ICU and hospital stays are prolonged in patients with hypoxemia^7–11^. Therefore, risk stratification for hypoxemia at the time of admission is important in the management of AAS.

While there are several reports of perioperative hypoxemia in patients with type-A AAS, there are only a few reports of hypoxemia in patients with type-B AAS^4–6^, and hypoxemia is difficult to predict in patients with type-B AAS. Several studies have reported that the peak level of C-reactive protein (CRP) is useful for predicting hypoxemia^4,12,13^. However, the peak CRP level is not known at the onset of AAS; thus, it cannot be used for risk stratification for hypoxemia at the time of admission. In other words, prediction of hypoxemia using factors that can be assessed immediately on admission is currently poorly known. Therefore, we evaluated predictive factors for hypoxemia in patients with type-B AAS based on their clinical variables at the time of admission.

## Methods

### Study participants

We retrospectively enrolled patients with type-B AAS hospitalized in Saiseikai Kumamoto Hospital, Japan from January 2012 to April 2020. Type B-AAS was diagnosed based on computed tomography (CT) with contrast enhancement. The following categories of patients were excluded: emergent surgery within 24 h of onset, onset time unknown, diagnosis later than 7 days after onset, acute heart failure, and infectious pneumonia. In previous studies, almost all hypoxemia events have been within 7 days, and about 80% of them have occurred within 3 days^6,12^. Therefore, to avoid underestimating hypoxemia events, we excluded patients who had diagnosis later than 7 days after onset.

### Evaluation and definition of clinical variables

We collected the basic characteristics of patients from their medical records, including age, sex, coexisting disease, laboratory data at admission and within 7 days after admission, CT scan findings, and ICU or hospital stay. Hypertension was defined as systolic blood pressure ≥ 140 mmHg and/or diastolic blood pressure ≥ 90 mmHg before onset or receiving antihypertensive medication. Diabetes mellitus was defined as glycated hemoglobin ≥ 6.5% or receiving oral medication for diabetes mellitus or insulin therapy. Dyslipidemia was defined as casual total cholesterol > 220 mg/dL or receiving medication for dyslipidemia. Obesity was defined as body mass index (BMI) ≥ 25 kg/m^2^. Because of the differences in body size between Japanese and Westerners, the definition of obesity used in this study was specific to the Japanese population, not the WHO definition. Peak CRP level was defined as the first peak after admission. In this study, we defined AAS as classic aortic dissection with a patent false lumen and intramural hematoma (IMH).

### Hypoxemia after type-B AAS

We defined hypoxemia after the onset of type-B AAS as a PaO_2_/FiO_2_ (P/F) ratio ≤ 200^14^ within 7 days (168 h) after onset. The estimated PaO_2_ level was adopted as the PaO_2_ value in patients without an arterial blood gas test^15^. We applied the estimated FiO_2_ level as the FiO_2_ level in patients not receiving invasive or non-invasive mechanical ventilation^16^.

### Statistical analysis

Continuous variables are presented as mean ± standard deviation for continuous variables with normal distribution, as median (interquartile range) for continuous variables with non-normal distribution, and as numbers (percentages) for categorical variables. We compared 2 variables with the Mann–Whitney U-test for continuous variables and the Fisher exact test for categorical variables. We used multivariate logistic regression analysis to clarify the factors associated with hypoxemia. For the multivariate model, we included clinical factors that had been reported as risk factors for hypoxemia in previous studies or were statistically significant. A receiver operating characteristics (ROC) curve analysis was performed to determine the cutoff value of significant variables for the prediction of hypoxemia. The predictive score was constructed based on the logistic model. The ROC analysis was also used for the risk score for hypoxemia. The factors were weighted based on the odds ratio (OR) by multivariate analysis performed with independent risk factors for hypoxemia. A *p* value < 0.05 was considered statistically significant. We used R version 3.1.1 for statistical analyses.

### Ethics approval and consent to participate

The study protocol was approved by the ethics committee of Saiseikai Kumamoto Hospital (approval number 907). Written informed consent was waived given the retrospective nature of the study by the ethics committee of Saiseikai Kumamoto Hospital.

## Results

### Baseline characteristics

**Figure 1:**
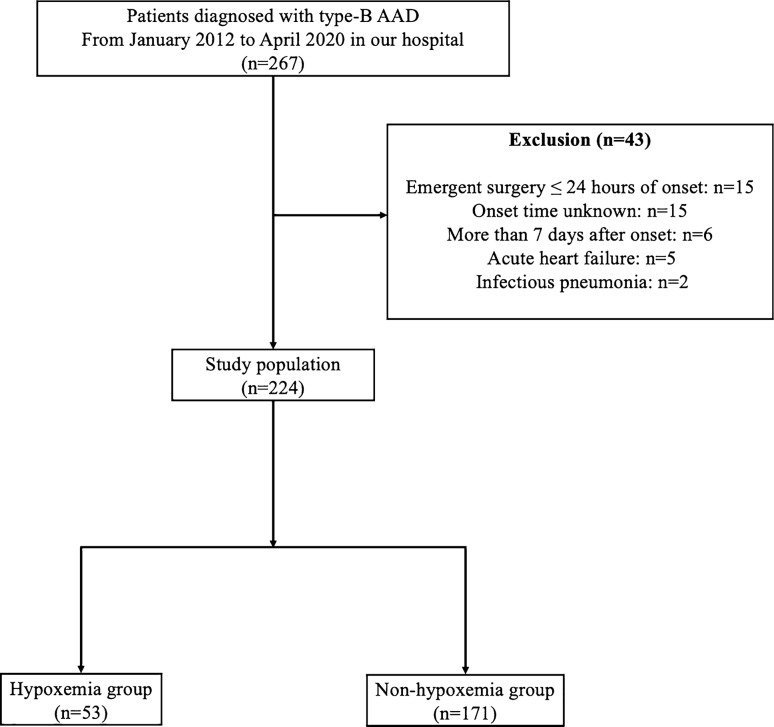
Flow diagram of study patients.

**Figure 2:**
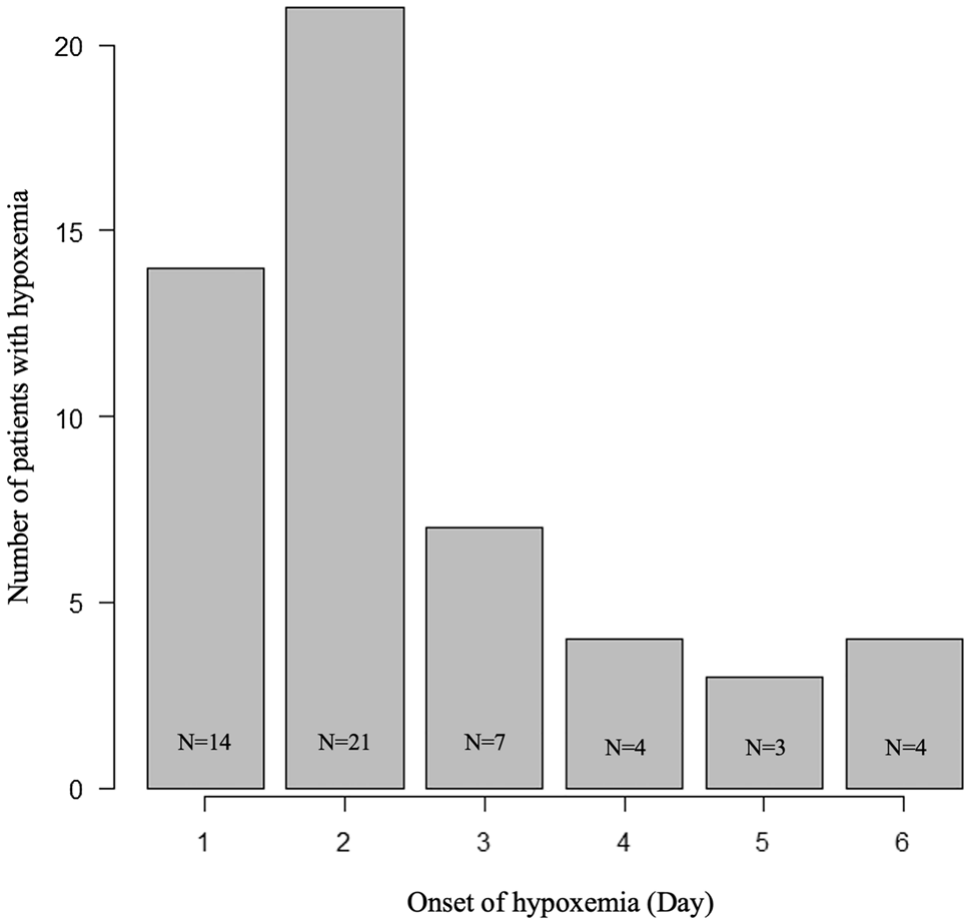
Number of patients and time from the onset of acute aortic syndrome to hypoxemia.

Among the 267 patients with type-B AAS enrolled, 43 were excluded due to emergent surgery within 24 h of onset (n = 15), onset time unknown (n = 15), more than 7 days after onset (n = 6), acute heart failure (n = 5), and infectious pneumonia (n = 2) (Fig. 1). Two hundred twenty-four patients (140 males, mean age 70 ± 14 years) were ultimately enrolled for further analyses. During hospitalization, 53 patients (23.7%) had hypoxemia (Hypoxemia group). The median time of detection of hypoxemia was 38 (23–58) hours from onset of AAS. Most of the hypoxemia was observed within 48 h (66.0%) (Fig. 2). Baseline characteristics of patients with type-B AAS are summarized in Table 1. The patients in the hypoxemia group had a significantly higher BMI compared with those in the non-hypoxemia group. Obesity, male sex, chronic kidney disease, and current smokers were significantly more common in the hypoxemia group than in the non-hypoxemia group. There were significantly more patients with patent false lumen and significantly fewer patients with IMH in the hypoxemia group compared with the non-hypoxemia group. Patients with hypoxemia had significantly higher levels of D-dimer, creatinine, and white blood cells at the time of hospitalization compared with patients without hypoxemia. After hospitalization, the patients with hypoxemia had significantly higher peak CRP levels than those without hypoxemia.

**Table 1:** Baseline characteristics of study participants with type-B acute aortic syndrome.

	Total (N = 224)	Hypoxemia (N = 53)	Non-hypoxemia (N = 171)	*p* value
Age	70.5 ± 13.8	67.3 ± 14.5	71.5 ± 13.5	0.057
Male	140 (62.5)	44 (83.0)	96 (56.1)	< 0.001
Body mass index (kg/m^2^)	23.4 (21.0–25.9)	24.8 (23.4–27.4)	22.9 (20.5–25.3)	< 0.001
Obesity	74 (33.0)	25 (47.2)	49 (28.7)	0.018
Current smoker	65 (29.0)	22 (41.5)	43 (25.1)	0.022
Hypertension	176 (78.6)	44 (83.0)	132 (77.2)	0.45
Diabetes mellitus	18 (8.0)	3 (5.7)	15 (8.8)	0.77
Dyslipidemia	71 (31.7)	22 (41.5)	49 (28.7)	0.088
Thoracic/abdominal aortic aneurysm	56 (25.0)	11 (20.8)	45 (26.3)	0.47
**Medications on admission**				
Antiplatelet agent	34 (15.2)	5 (9.4)	29 (17.0)	0.27
Anticoagulant	7 (3.1)	2 (3.8)	5 (2.9)	0.67
Antihypertensive treatment	115 (51.3)	24 (45.3)	91 (53.2)	0.35
**Vital signs on admission**				
Systolic blood pressure (mmHg)	160 (145–188)	173 (148–191)	163 (144–184)	0.13
Diastolic blood pressure (mmHg)	85 (74–98)	88 (74–108)	84 (74–96)	0.37
Heart rate (/min)	77 ± 13	78 ± 12	76 ± 14	0.42
**Laboratory data on admission**				
White blood cell (/µL)	9100 (7275–11,625)	9799 (8200–13,200)	8800 (6800–10,800)	1.5 × 10^–3^
Hemoglobin (g/dL)	13.3 (12.0–14.8)	14.2 (12.7–15.1)	13.2 (11.9–14.4)	0.068
D-dimer (µg/mL)	11.1 (5.8–25.9)	14.5 (6.5–37.2)	6.1 (2.3–13. 8)	< 0.001
Creatinine (mg/dL)	0.82 (0.65–1.12)	1.03 (0.82–1.34)	0.79 (0.63–1.04)	< 0.001
CRP (mg/dL)	0.22 (0.07–0.75)	0.23 (0.08–0.42)	0.22 (0.07–0.82)	0.59
BNP (pg/mL)	39 (19–87)	44.5 (22–74)	37 (19–90)	0.93
**CT findings on admission**				
Patent false lumen	92 (41.1)	33 (62.3)	59 (34.5)	< 0.001
IMH	132 (58.9)	20 (37.7)	112 (65.5)	< 0.001
Maximum aneurysm diameter (mm)	37 (33, 41)	38 (35, 40)	36 (32, 41)	0.14
Pleural effusion	45 (20.1)	15 (28.3)	20 (17.5)	0.12
COPD	34 (15.2)	5 (9.4)	29 (17.0)	0.064
Interstitial pneumonia	4 (1.8)	0 (0.0)	4 (2.3)	0.58
**After hospitalization**				
Minimum PaO_2_/FiO_2_ ratio	267 (209–328)	160 (133–175)	305 (246–346)	< 0.001
Timing of hypoxemia (hours)	–	38 (23, 58)	–	–
Peak CRP level (mg/dL)	10.5 (6.8,-5.7)	13.8 (10.5–23.3)	9.1 (6.0–13.1)	< 0.001
Timing of peak CRP level (days)	4 (3, 6)	3 (3, 5)	4 (3, 6)	0.43
ICU stay (days)	5 (3–8)	7 (5–10)	5 (3–7)	< 0.001
Hospital stay (days)	16 (13–22)	20 (14–26)	16 (12.5–21)	0.039

Values are mean ± standard deviation, median (interquartile range), or n (%).

*CRP* C reactive protein, *BNP* brain natriuretic peptide, *CT* computed tomography, *IMH* intramural hematoma, *COPD* chronic obstructive pulmonary disease, *ICU* intensive care unit.

Among patients with type-B AAS, the hypoxemia group had longer duration of hospitalization compared with the non-hypoxemia group (median 20 vs. 16 days, respectively; *p* = 0.039) (Table 1). In addition, compared with patients in the non-hypoxemia group, those with hypoxemia had a longer ICU stay (median 5 vs. 7 days, respectively; *p* < 0.001) (Table 1).

### Predictors of in-hospital hypoxemia in patients with type B-AAS

The factors of male sex, obesity, current smoker, high D-dimer level, and patent false lumen were associated with hypoxemia by univariate analysis (Table 2). In the multivariate logistic analysis, male sex (OR 2.87; 95% confidence interval [CI] 1.24–6.63; *p* = 0.014), obesity (OR 2.36; 95% CI 1.13–4.97; *p* = 0.023), patent false lumen (OR 2.33; 95% CI 1.09–4.99; *p* = 0.029), and high D-dimer levels (OR 1.01; 95% CI 1.00–1.02; *p* = 0.047) were significantly associated with a higher rate of hypoxemia (Table 2).

**Table 2:** Univariate and multivariate logistic regression analysis of in-hospital hypoxemia.

	Univariate			Multivariate		
	OR	95% CI	*p* value	OR	95% CI	*p* value
Age	0.98	0.96–1.00	0.056	1.00	0.97–1.03	0.91
Male	3.82	1.75–8.32	< 0.001	2.87	1.24–6.63	0.014
Obesity	2.27	1.20–4.29	0.0012	2.36	1.13–4.97	0.023
Current smoker	2.26	1.18–4.34	0.015	1.35	0.62–2.95	0.46
Diabetes mellitus	0.64	0.18–2.30	0.49	–	–	–
TAA/AAA	0.73	0.35–1.55	0.42	–	–	–
Antiplatelet agent	0.51	0.19–1.39	0.19	–	–	–
Anticoagulant	1.30	0.25–6.91	0.76	–	–	–
Antihypertensive treatment	0.73	0.39–1.35	0.31	–	–	–
Systolic blood pressure	1.01	0.99–1.02	0.10	–	–	–
Diastolic blood pressure	1.01	0.99–1.03	0.07	–	–	–
Heart rate	1.01	0.99–1.03	0.41	–	–	–
WBC count > 15,000/µL	1.68	0.55–5.14	0.37	0.56	0.13–2.47	0.44
Hemoglobin	1.15	0.99–1.34	0.070			
D-dimer	1.01	1.00–1.02	5.9 × 10^−3^	1.01	1.00–1.02	0.047
Creatinine	1.05	0.87–1.25	0.62	–	–	–
BNP	0.99	0.99–1.00	0.41	–	–	–
CRP	0.93	0.83–1.04	0.19	–	–	–
Patent false lumen	3.13	1.65–5.93	< 0.001	2.33	1.09–4.99	0.029
Intramural hematoma	0.62	0.31–1.24	0.18	–	–	–
Maximum aneurysm diameter	1.02	0.98–1.06	0.36	–	–	–
Pleural effusion	1.93	0.94–3.96	0.072	–	–	–
COPD	0.51	0.19–1.39	0.19	–	–	–
Peak CRP	1.12	1.07–1.17	< 0.001	–	–	–

*CI* confidence interval, *OR* odds ratio, *TAA* thoracic aortic aneurysm, *AAA*: abdominal aortic aneurysm, *WBC* white blood cell, *CRP* C reactive protein, *BNP* brain natriuretic peptide, *COPD* chronic obstructive pulmonary disease.

### Predicting the score for hypoxemia in patients with type-B AAS

**Figure 3:**
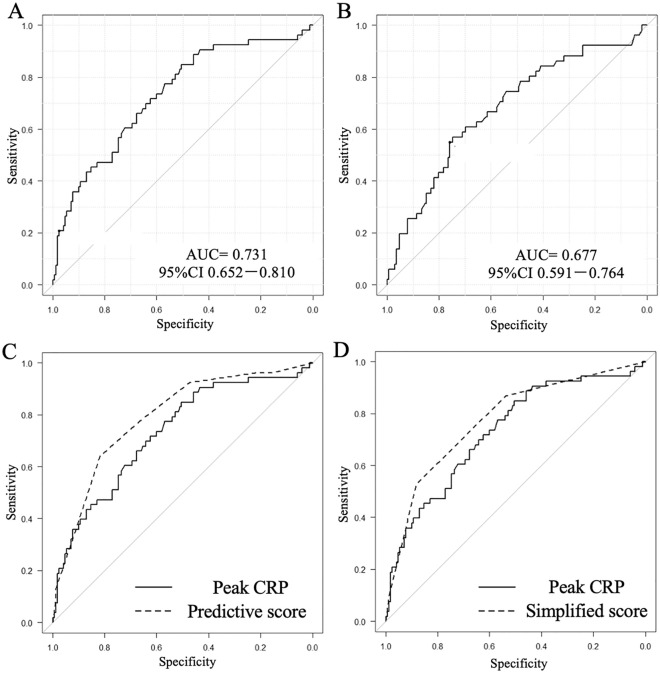
Receiver operating characteristic (ROC) curve of peak C-reactive protein level (**A**) and D-dimer level (**B**) for in-hospital hypoxemia in patients with type-B acute aortic syndrome. Comparison of the ROC curve between the peak CRP level (AUC 0.731; 95% CI 0.652–0.81) and the predictive score for hypoxemia (AUC 0.791; 95% CI 0.723–0.859) (**C**). Comparison of the ROC curve between the peak CRP level (AUC 0.731; 95% CI 0.652–0.81) and the simplified score for predicting hypoxemia (AUC 0.777; 95% CI 0.707–0.847) (**D**).

**Figure 4:**
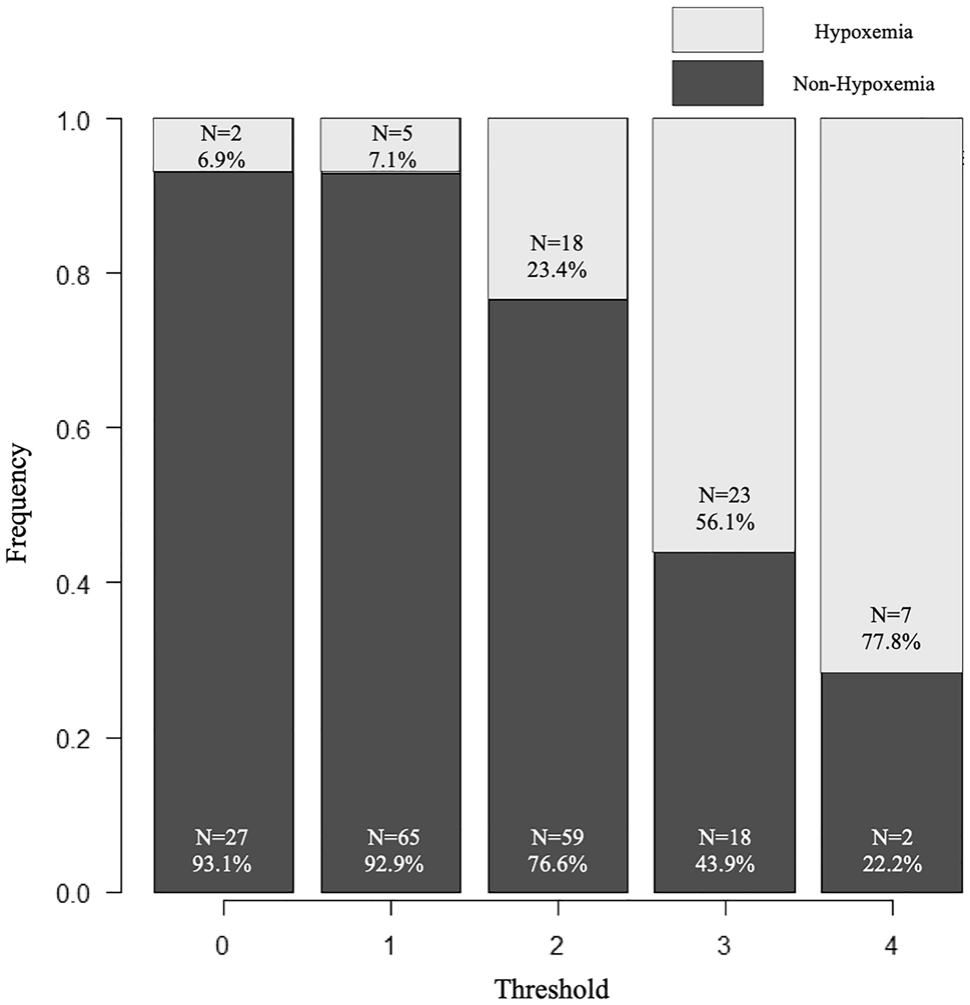
Agreement between the threshold of simplified score and the observed frequency.

As reported previously, peak CRP level was also a predictor of hypoxemia in the present analysis (OR 1.12; 95% CI 1.07–1.17; *p* < 0.001). In ROC curve analysis, the area under the curve (AUC) of the peak CRP level was also favorable (AUC, 0.731; 95% CI 0.652–0.81) (Fig. 3A). However, because we could not determine the peak CRP level at the time of admission, we evaluated each predictive score with the variables collected at admission. In the ROC curve analysis for hypoxemia, the optimal cutoff value of D-dimer for predicting hypoxemia was ≥ 14.5 µg/mL, with a sensitivity of 54.9% and a specificity of 75.9% (Fig. 3B). In addition, the median value of D-dimer at admission was also 14.5 µg/mL. In the multivariate logistic analysis that we performed again with D-dimer level ≥ 14.5 µg/mL as one factor, male sex (OR 3.61; 95% CI 1.55–8.42; *p* = 0.003), obesity (OR 3.74; 95% CI 1.67–8.39; *p* = 0.0013), patent false lumen (OR 2.09; 95% CI 1.03–4.27; *p* = 0.042), and D-dimer level ≥ 14.5 µg/mL (OR 5.68; 95% CI 2.52–12.8; *p* < 0.001) were significantly associated with higher rates of hypoxemia. Based on the OR by multivariate analysis, male sex, obesity, patent false lumen, and D-dimer level ≥ 14.5 µg/mL were scored as 4, 4, 2, and 6 points, respectively. The AUC of the predictive score was 0.791 (95% CI 0.723–0.859). For application to clinical practice, we analyzed a simplified score for predicting hypoxemia, in which all factors are scored as 1 point each. As a result, the AUC of the simplified score was 0.777 (95% CI 0.707–0.847). In the ROC curve analysis, an AUC of 0.7–0.9 indicates moderate accuracy^17^, suggesting similarity between the AUC of peak CRP and the two predictive scores (Fig. 3C, D). The predictive score (range 0–16) was able to predict hypoxemia with a sensitivity of 64.2% and a specificity of 81.9% if the score was 10 points or higher. Similarly, an optimal cutoff value for the simplified score (range 0–4) in predicting hypoxemia was 3 points or higher, with a sensitivity of 52.8% and a specificity of 88.3%. The distribution (Fig. 4), sensitivity, specificity, positive predictive value, and negative predictive value are shown for each level of the simplified score for predicting hypoxemia (Table 3).

**Table 3:** Simplified score for predicting hypoxemia in patients with type-B acute aortic syndrome.

Threshold	≥ 1	≥ 2	≥ 3	≥ 4
Sensitivity (%)	96.2	86.8	52.8	9.4
Specificity (%)	15.8	53.8	88.3	98.8
Positive predictive value (%)	26.5	37.2	55.8	71.2
Negative predictive value (%)	92.9	92.8	85.6	77.5

### Clinical events in patients with type-B AAS

At the initial first hospitalization, several clinical events occurred in the participants of this study. In-hospital death, aortic rupture, malperfusion, and aortic enlargement were observed in 8, 2, 5, and 13 patients, respectively (Table 4). In-hospital mortality was significantly higher in the hypoxemia group than in the non-hypoxemia group (9.4% vs. 1.8%; *p* = 0.020). On the other hand, the mean hospital and ICU stay for patients who had in-hospital death was 3.4 and 3.4 days in the hypoxemia group and 3.3 and 9.7 days in the non-hypoxemia group. In addition, seven of the patients who had these events underwent interventions in the course of their hospitalization. Four patients underwent surgical procedures and three underwent endovascular interventions.

**Table 4:** Clinical events in patients with type-B acute aortic syndrome.

	Total (N = 224)	Hypoxemia (N = 53)	Non-hypoxemia (N = 171)	*p* value
In-hospital death	8 (3.6)	5 (9.4)	3 (1.8)	0.020
Rupture of aorta	2 (0.9)	1 (1.9)	1 (0.6)	0.42
Malperfusion	5 (2.2)	3 (5.7)	2 (1.2)	0.088
Aortic enlargement	13 (5.8)	1 (1.9)	12 (7.0)	0.31
Intervention (surgery or endovascular)	7 (3.1)	2 (3.8)	5 (2.9)	0.67

Values are median (interquartile range), or n (%).

## Discussion

In present study, male sex, obesity, patent false lumen, and high D-dimer level were independent predictors of hypoxemia by multivariate analysis. In addition, the peak CRP level was significantly associated with hypoxemia by univariate analysis. The four clinical variables (male sex, obesity, patent false lumen, and D-dimer level ≥ 14.5 µg/mL) and peak CRP level had comparable ROC curves. This study showed that patients with type-B AAS with in-hospital hypoxemia (23.9%) had a longer hospital stay and ICU stay compared with those in the non-hypoxemia group. These results demonstrate characteristics, predictors, and disadvantages of hypoxemia in patients with type-B AAS.

In this study, patients with hypoxemia had a longer hospital stay and ICU stay compared with patients without hypoxemia. Several studies have also reported that lengths of stay in the ICU and the hospital were longer in patients with hypoxemia^7–11^. Prolonged stay in the ICU may be because mechanical ventilation, non-invasive positive pressure ventilation, and nasal high-flow systems are often required in patients with type-B AAS with hypoxemia. Prolonged hospital stay may be because hypoxemia prevents rehabilitation from progressing and makes it difficult to investigate the effects of exertion on blood pressure elevation.

In previous reports, the incidence of hypoxemia was reported to be 39–49% in type-B and 28.6–46.5% in type-A AAS^5–7,10^, with no significant difference between types. However, the low rate found in this report may be related to the fact that the number of patients in this study is higher than in past reports. The underlying mechanism of hypoxemia in AAS is not yet known, but in previous reports, it has been attributed to systemic inflammation^4,18–20^. Elevated CRP level, an inflammatory marker, has been reported to be related to lung oxygenation impairment in patients with AAS^5,12,13,18,19^. Those studies explained that the CRP level is correlated with hypoxemia through the production of inflammatory cytokines, such as interleukin (IL) -6 and IL-8. Lung oxygenation impairment in patients with AAS may develop by the same mechanism as in acute respiratory distress syndrome (ARDS)^4,18^. In fact, the peak CRP level after hospitalization was associated with the development of hypoxemia in the present study. However, imaging findings often do not show typical pulmonary edema as in ARDS. Therefore, other causes of hypoxemia may also coexist. Several studies have reported lung ventilation-perfusion (V/Q) mismatch due to hypoxic pulmonary vasoconstriction and have demonstrated that nitric oxide inhalation was effective in preventing hypoxemia^21,22^. In addition, they have speculated that an intrapulmonary shunt may be present due to an excessive inflammatory response.

Several reports have identified obesity as a risk for hypoxemia in AAS^6,8–10,23^. Because elevations of IL-1β, TNF-α, and IL-6 in obese patients with AAS have been observed^8^, they may induce lung injury and also promote the release of inflammatory factors. Furthermore, obese patients have chronic inflammation and oxidative stress, which may result in direct damage to the cellular membranes and the release of vasoactive substances^9,24,25^. Obesity is an important risk factor for hypoxemia and ventilation in the ICU^26^. Since airway closure and atelectasis are facilitated by the decreased functional residual capacity among patients with obesity, obesity-induced atelectasis may contribute to the V/Q mismatch. In addition, activity limitations in the management of AAS may accelerate atelectasis.

In the present analysis, a patent false lumen was one of the risk factors for hypoxemia. However, there is a discrepancy in the classification of aortic dissection between Japan and Europe or the United States^27–29^. This is especially true for the definition and classification of IMH. In Europe and the United States, IMH is defined as the absence of a patent false lumen or tear. However, the Japanese guideline states that it is difficult to differentiate individuals with/without a tear from imaging findings alone, and all cases are often treated as aortic dissection in Japan^29^. In our study, we used the CT classification of classical aortic dissection (patent false lumen) and IMH (non-patent false lumen). Previous reports have revealed that the size of the false lumen is a predictor of hypoxemia in type-B AAS, regardless of whether the false lumen is patent or not^5^. Also, the D-dimer value has been reported to be correlated positively with the CRP level and the anatomic extent of aortic dissection^30^. Kuehl et al.^31^ demonstrated that vessel wall inflammation was identified by positron emission tomography in patients with AAS. Therefore, the greater the extent of aortic dissection, the stronger the systemic inflammatory response is likely to be. In other words, an elevated d-dimer level at admission, which was identified as a risk factor in this study, may be a surrogate marker for inflammation in patients with type-B AAS. Alternatively, it may reflect a V/Q mismatch due to microthrombosis in the pulmonary capillaries.

WBC count > 15,000/µL and current smoker, previously reported as risk factors for hypoxemia in patients with AAS^7,11^, were not associated with hypoxemia in the present study. This result may be influenced by the fact that our study had a lower incidence of current smokers (n = 15, 6.7%) and lower WBC counts compared with past reports. As for current smokers, it was reported in patients with type-A AAS, which may reflect a risk factor for hypoxemia after cardiac surgery^32^. However, because we wanted to minimize the effects of surgery, we excluded all emergency surgery cases in this study. Therefore, current smoker may not have been identified as a risk factor. On the other hand, although male sex was a predictor for hypoxemia in our analysis, there have been no such reports in the past; rather, there was a report that female sex was a risk factor^9^. Our study contained relatively more women (37.5%) than the past studies (15–25%)^4,5,7,11^, which may have highlighted the proportion of hypoxemia in men. In addition, women develop aortic dissection an average of 6–7 years later than men^33^, and the onset at a younger age may be related to a high degree of inflammation. We must await the results of future studies on gender differences in patients with AAS.

In past reports on the risk of hypoxemia, the peak CRP level was most frequently cited as a predictor^5,12,13,18,19^. However, the peak CRP level after hospitalization is not known at the time of admission. Therefore, it cannot be used clinically as a predictor, even if it helps to identify the mechanism. Actually, in the present study, most hypoxemia occurred on days 1–2, whereas the median peak CRP level was on day 4 (interquartile range; 3–6), indicating that hypoxemia occurs first. Therefore, we focused on the evaluation at admission in our study. This study showed that four factors (male sex, obesity, patent false lumen, and D-dimer level ≥ 14.5 µg/mL) that can be evaluated at the time of admission could be used to predict hypoxemia, similar to peak CRP level, on the basis of the ROC curves. Furthermore, since we can evaluate the score using only four simple factors, this can contribute to stratification of hypoxemia risk in primary medical settings. When the cutoff values were set at 3 points or greater, the scores were able to predict the occurrence of hypoxemia in patients with type-B AAS with a high predictive value. Patients with a higher score can be identified as those who require more restrictive management in the ICU. Vigilance in identifying these patients may allow for earlier diagnosis of hypoxemia and better management of type-B AAS. In addition, hypoxemia after hospitalization in the presence of a lower score is more likely to be due to other causes and may require more scrutiny.

This study has several limitations: first, it was a retrospective single-center study, and the utility of the predictive score has not been examined in other populations. The peak CRP test was carried out at the discretion of the physician and was not performed daily. However, the appropriate period for follow-up tests is not clearly established in patients with AAS. Finally, predictive values were also used for both PaO_2_ and FiO_2_. Therefore, a further large-scale prospective study is required to strengthen the validity of the present results.

## Conclusions

Hypoxemia after the onset of type-B AAS was associated with a longer ICU stay and a longer hospital stay. The present study showed that male sex, obesity, patent false lumen, and high D-dimer levels could be significantly associated with hypoxemia in patients with type-B AAS. Therefore, we should pay more attention to patients with these clinical factors, although further study is necessary to confirm our results.

## Data Availability

The datasets used and/or analyzes in the current study are available from the corresponding author upon reasonable request.

## Abbreviations

AAA: Abdominal aortic aneurysm
AAS: Acute aortic syndrome
ARDS: Acute respiratory distress syndrome
AUC: Are under the curve
BMI: Body mass index
BNP: Brain natriuretic peptide
CI: Confidence interval
COPD: Chronic obstructive pulmonary disease
CRP: C reactive protein
CT: Computed tomography
ICU: Intensive care unit
IL: Interleukin
IMH: Intramural hematoma
OR: Odds ratio
P/F: PaO_2_/FiO_2_
ROC: Receiver operating characteristic
TAA: Thoracic aortic aneurysm
ULP: Ulcer-like projection
V/Q: Ventilation-perfusion
WBC: White blood cell
